# A real-world data validation of the value of early-stage SIR modelling to public health

**DOI:** 10.1038/s41598-023-36386-9

**Published:** 2023-06-06

**Authors:** Taoran Liu, Jian Huang, Zonglin He, Yin Zhang, Ni Yan, Casper J. P. Zhang, Wai-Kit Ming

**Affiliations:** 1 Department of Infectious Diseases and Public Health, Jockey Club College of Veterinary Medicine and Life Science, City University of Hong Kong, Hong Kong, SAR China; 2 Department of Epidemiology and Biostatistics, School of Public Health, St Mary’s Campus, Imperial College London, London, UK; 3 Singapore Institute for Clinical Sciences (SICS), Agency for Science, Technology and Research (A*STAR), Singapore, Singapore; 4 Division of Life Science, The Hong Kong University of Science and Technology, Hong Kong, China; 5 Department of Medicine, LKS Faculty of Medicine, The University of Hong Kong, Hong Kong, China; 6 Department of Public Health and Preventive Medicine, School of Medicine, Jinan University, Guangzhou, China; 7 School of Public Health, LKS Faculty of Medicine, The University of Hong Kong, Hong Kong, China

**Keywords:** Infectious diseases, Health policy, Public health, Epidemiology

## Abstract

Performance of Susceptible-Infected-Recovered (SIR) model in the early stage of a novel epidemic may be hindered by data availability. Additionally, the traditional SIR model may oversimplify the disease progress, and knowledge about the virus and transmission is limited early in the epidemic, resulting in a greater uncertainty of such modelling. We aimed to investigate the impact of model inputs on the early-stage SIR projection using COVID-19 as an illustration to evaluate the application of early infection models. We constructed a modified SIR model using discrete-time Markov chain to simulate daily epidemic dynamics and estimate the number of beds needed in Wuhan in the early stage of COVID-19 epidemic. We compared eight scenarios of SIR projection to the real-world data (RWD) and used root mean square error (RMSE) to assess model performance. According to the National Health Commission, the number of beds occupied in isolation wards and ICUs due to COVID-19 in Wuhan peaked at 37,746. In our model, as the epidemic developed, we observed an increasing daily new case rate, and decreasing daily removal rate and ICU rate. This change in rates contributed to the growth in the needs of bed in both isolation wards and ICUs. Assuming a 50% diagnosis rate and 70% public health efficacy, the model based on parameters estimated using data from the day reaching 3200 to the day reaching 6400 cases returned a lowest RMSE. This model predicted 22,613 beds needed in isolation ward and ICU as on the day of RWD peak. Very early SIR model predictions based on early cumulative case data initially underestimated the number of beds needed, but the RMSEs tended to decline as more updated data were used. Very-early-stage SIR model, although simple but convenient and relatively accurate, is a useful tool to provide decisive information for the public health system and predict the trend of an epidemic of novel infectious disease in the very early stage, thus, avoiding the issue of delay-decision and extra deaths.

## Introduction

In late 2019, a novel coronavirus disease (COVID-19)^1^ outbreak caused by a pathogen later identified as severe acute respiratory syndrome coronavirus 2 (SARS-CoV-2)^2^ emerged in Wuhan, Hubei Province, China. Human-to-human transmission was confirmed by National Health Commission in China in January 2020^3^, and the virus has since spread over 218 countries with nearly 70 million confirmed cases by the end of 2020^4^. Societies are facing enormous challenges regarding health systems and economy. Many countries have witnessed exponential increases in the number of COVID-19 cases during the pandemic. Some has experienced multiple waves of outbreak^5–7^. As such, timely infectious disease control is crucial to safeguard public health and mitigate economic and social impacts. Therefore, infectious disease modelling to project the severity of a novel disease outbreak needs to be constructed to inform policymaking^8^.

Compartmental models have been widely used to predict the trend in the transmission of infectious diseases. For instance, several previous studies which aimed to model for SARS propagation^9,10^, predict the trend of SARS pandemic, have proved compartmental models to be the effective tool to provide essential information of infectious diseases and guidelines for implementation of containing measures. However, in the early stage of a novel disease outbreak, knowledge about the virus and transmission is limited, which result in a greater uncertainty of such modelling, and disease progress may be oversimplified using traditional compartmental models. Additionally, the reporting system and data release might not be updated timely due to limited testing capacity and other logistic issues^11,12^. Taken together, the application^13^ of compartmental models in the early stage of epidemic needs further validation.

In this study, we aimed to evaluate the application of compartmental models in the early stage of a novel infectious disease outbreak. Specifically, we constructed modified SIR models based on data at different stages of the COVID-19 epidemic in Wuhan and investigated the impact of model input on the projection. We also compared the early-stage SIR projections with real-world data (RWD).

## Methods

### Data resources

We obtained the daily number of newly confirmed cases, cumulative number of confirmed cases, daily number of new deaths, cumulative number of deaths, daily number of new recoveries and cumulative number of recoveries in both Wuhan city and Hubei province from January 10, 2020 to March 30, 2020 from the Outbreak Notification of National Health Commission (NHC) of People’s Republic of China^14,15^. Challenges we faced were the accessibility of existing cases in ICU, therefore, we included existing number of cases in ICU only in Hubei province^15^ from January 10, 2020 to March 30, 2020 in our further study. We estimated the actual daily number of beds needed in isolation ward and ICU by subtracting the cumulative number of deaths and recoveries from the cumulative number of confirmed cases.

### Model construction

In our modified SIR model, we divided the whole population of Wuhan (11 million) into three compartments^16–19^, i.e., susceptible (S), infectious (I) and removal (R). Individuals who die of or recover from the infection would be moved into the removal compartment and we assume recovered individuals would have acquired immunity^20,21^. Specially, we constructed a SIR model using discrete-time Markov chain^22–24^ to simulate daily epidemic dynamics and transition between different compartments to predict the burden on the public health system in the early stage of the COVID-19 epidemic in Wuhan. In the discrete-time Markov chain, one cycle in our model represents one day in actual time. Given that early investigation has suggested human-to-human transmission occurred among close contacts since the mid-December 2019^25^, we set the first day of our model on 15th December 2019. Since we aimed to investigate the application of SIR model in the early stage of an epidemic, we ended our projection on 31st March 2020, when the peak of epidemic has passed, and the daily number of newly confirmed cases dropped to a relatively low level.

### Parameters and scenarios

The parameters fed into the SIR model represent the transition probabilities from one status to another within each cycle (Table 1). In our analysis, we considered eight scenarios based on the progression of the epidemic. Different scenarios used parameters estimated based on the data available at different stages of the epidemic (from 100 to 12,800 cumulative confirmed cases). The parameters affected by the scenarios are “DailyNewCase.Rate”, “DailyRemoval.Rate” and “ICU.Rate”. “DailyNewCase.Rate” was estimated based on daily number of newly confirmed cases in Wuhan city, while “DailyRemoval.Rate” and “ICU.Rate” were estimated based on daily number of removal cases, daily number of existing confirmed cases and daily number of existing ICU cases in Hubei province since such data were not released at the city level.

**Table 1:** Parameters, interpretation, and sources included in SIR Model.

Sources	Parameters^a^	Base-case value
Population census	Population (Wuhan)	11 million
National Health Commission	Number of daily new confirmed cases (Wuhan)	Average number of new confirmed cases of the time periods = $\frac{{Sum\, of\,\, daily\,\,new\,\, confirmed \,\,cases \left( {Wuhan} \right)}}{Days\,\, in \,\,the\,\,periods}$
	Beds needed in isolation ward and ICU (RWD)	*Cumulative confirmed cases -cumulative deaths-cumulative-cumulative recoveries*
	Number of daily existing confirmed cases (Wuhan)	Average number of existing confirmed cases of the time periods = $\frac{{Sum\,\, of\,\,Beds\,\, needed\,\, in\,\, isolation\,\,ward\,\,and \,\,ICU\,\,\left( {Wuhan} \right)}}{Days\,\,in\,\, the\,\,periods}$
	Number of daily existing confirmed cases (Hubei)	Average number of existing confirmed cases of the time periods = $\frac{{Sum\,\, of\,\,Beds\,\, needed\,\,in\,\,isolation \,\,ward\,\,and\,ICU\,\, \left( {Hubei} \right)}}{Days\,\, in \,\,the\,\,periods}$
	Number of daily existing ICU (Hubei)	Average number of existing ICU cases of the time periods = $\frac{{Sum\,\,of\,\, existing\,\, ICU\,\,\left( {Hubei} \right)}}{Days\,\, in\,\, the \,\,periods}$
	Number of daily removal (Hubei)	Average number of daily removal cases = $\frac{Sum\,\, of\,\, number\,\,of\,\, daily\,\,new\,\,death\, + \,Sum \,\,of\,\, deaily\,\, new\,\, recovery}{{Days \,\,in \,\,the\,\,periods}}$
	DailyNewCase.Rate (Based on Wuhan)	Rate of infectious individuals being diagnosed from total population = $\frac{{Number\,\,of\,\,daily\,\,new\,\, confirmed\,\,cases\,\,\left( {Wuhan} \right)}}{{Population\,\,\left( {Wuhan} \right)}}$
	DailyRemoval.Rate (Based on Hubei)	Rate of existing cases being death or recovery = $\frac{{Number\,\,of\,\,daily\,\,removal\,\,\left( {Hubei} \right)}}{{Number \,\,of\,\,existing\,\,confirmed\,\,cases\,\,\left( {Hubei} \right)}}$
	ICU.Rate (Based on Hubei)	Ratio of daily existing ICU cases to daily number of existing cases = $\frac{{Number\,\, of\,\, daily\,\, existing\,\, ICU\,\, \left( {Hubei} \right)}}{{Number\,\, of\,\, daily\,\, existing\,\, confirmed\,\, cases\,\, \left( {Hubei} \right)}}$
Published research	Risk of fatality among hospitalised cases^b^	14%
Assumptions	Dx.Rate	Assuming 50%, 70%, 90% of the infected population can be accurately diagnosed
	Days undiagnosed	Days for undiagnosed cases to recover = 12.5 days
	Self.Recovery.Rate	Rate of self-recovery for individuals who were undiagnosed = $\frac{1}{Days\,\,undiagnosed}$
	UnDx2Case.Rate	Days for undiagnosed cases switching to confirmed cases = $\frac{Dx.Rate}{{Days\,\,undiagnosed}}$
	Public health efficacy	Assuming 30%, 50%, 70%, 90% efficacy of public health intervention

*ICU* intensive care unit, *RWD* Real-world data.

^a^All probabilities are probabilities within one cycle in the model.

^b^Wu et al. Real-time tentative assessment of the epidemiological characteristics of novel coronavirus infections in Wuhan, China, as on 22 January 2020. Eurosurveillance.

Specifically, Scenario 1 estimated these parameters based on data on the day when the reported cumulative confirmed cases reached 100; Scenario 2 estimated these parameters based on data between the day of 100 and the day of 200 cumulative confirmed cases; Scenario 3 estimated these parameters based on data between the day of 200 and the day of 400 cumulative confirmed cases; and the remaining five scenarios were constituted by the intervals in which the number of cumulative confirmed cases doubled (Supplementary Table 1). Additionally, we performed a sensitivity analysis based on parameters estimated using data from the day of 800 to the day of 3200 cumulative confirmed cases.

Considering that undiagnosed infectious individuals would most likely experience mild symptoms, we hypothesized the average time needed for recovery (Days undiagnosed) for undiagnosed infectious individuals to be 12.5 days^26^ and the probabilities of self-recovering (Self.Recovery.Rate) without hospitalization within any given day to be 1/12.5. Additionally, we assumed three scenarios of diagnosis rate (Dx.Rate), i.e., 50%, 70%, and 90%. Therefore, the undiagnosed rate was calculated using diagnosis rate divided by ‘days undiagnosed’. An overall death rate of 14% among the hospitalized cases was used according to the investigation by researchers from the University of Hong Kong^27^. Additionally, to model the effect of public health intervention implemented in Wuhan, we assumed 30%, 50%, 70%, and 90% efficacy in each scenario when comparing to the RWD. All the model inputs of different scenarios are shown in Supplementary Table 1.

The number of beds needed in isolation ward was the sum of daily total isolation ward patients and daily total undiagnosed cases. While the number of beds needed in ICU was equal to the number of daily total ICU cases.

### Transition and Markov chain

**Figure 1:**
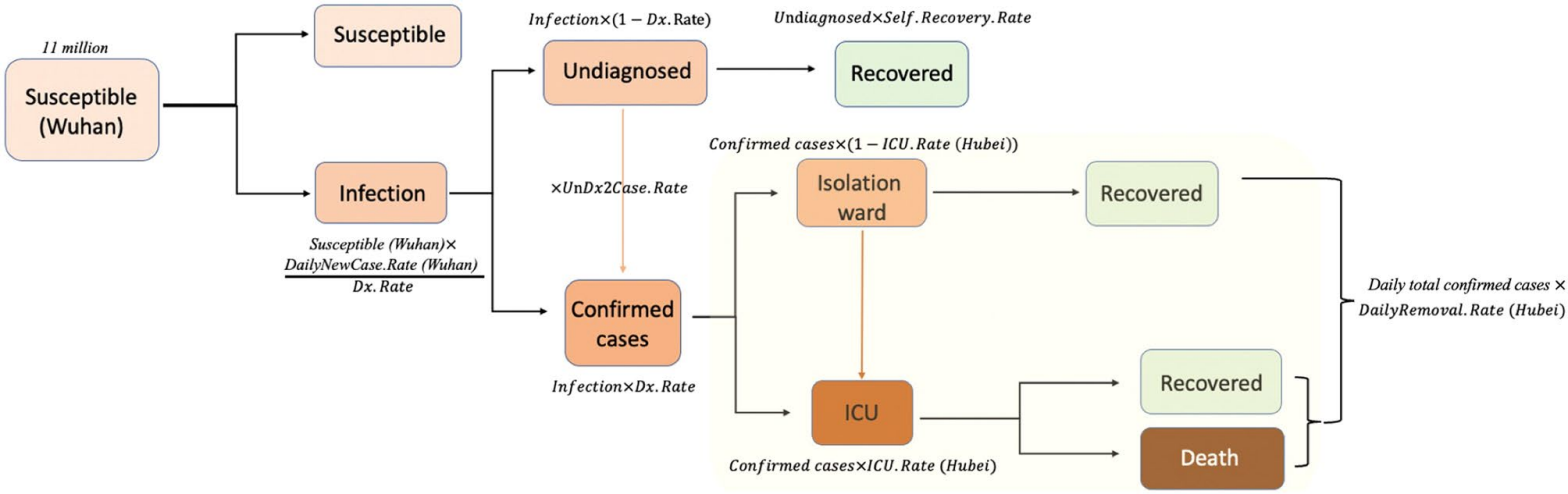
Simulation of COVID-19 epidemic dynamic in Wuhan using SIR model.

Specifically, in our model, as shown in Fig. 1, “susceptible” population in Wuhan is categorized into the susceptible compartment (S). When the susceptible become infected (I) with the rate of $\frac{{DailyNewCase.Rate\,\, \left( {Wuhan} \right)}}{Dx.Rate}$, they will be categorized into either the “undiagnosed” state with the rate of “1-*Dx.Rate*” or “confirmed cases” state with the rate of “*Dx.Rate”*. Meanwhile, some confirmed cases will be categorized into “isolation ward” state with the rate of “1-*ICU.Rate (Hubei)*”, and others will be categorized into “ICU” state with the rate of “*ICU.Rate (Hubei)*”.

Undiagnosed cased will remain undiagnosed and self-recover with rate of “Self.Recovery.Rate”, or become confirmed cases with rate of “*UnDxCase.Rate*”, and be admitted to isolation ward or ICU. Confirmed cases in the isolation ward will recover (R) or be admitted to ICU if their symptoms deteriorate. Confirmed cases in the ICU will recover (R) or die of the infection (R). We considered “Removal” as those recovered or dead from the health system. The total number of removals (recovered plus dead from the health system) should be calculated as total number of daily confirmed cases times the rate “DailyRemoval.Rate (Hubei)”.

Susceptible, infected and removal compartments are three discrete states that defined as state 0, 1 and 2. Assuming that, the number of individuals at any state and any time* t* is $X_{t}$, then $X_{t}$ should be the stochastic process of state 0, 1, 2. Then the first order time-homogeneous Markov dependency should be formulated as:

$$P\left( {X_{m} = i_{m} |X_{m - 1} = i_{m - 1} , \ldots , X_{1} = i_{1} , X_{0} = i_{0} } \right) = P\left( {X_{m} = i_{m} |X_{m - 1} = i_{m - 1} } \right)$$

### Comparison of SIR projection and RWD

We compared our projection of scenarios of different public health interventions to the RWD to validate the application of our model. Specifically, we calculated the Root Mean Square Errors (RMSEs) to check how our projection result fit the RWD. For each scenario, we calculated RMSE for the projection from the first day of which data for parameter estimation were available to the day of RWD peak (18th Feb 2020), e.g., RMSE for Scenario 1 was calculated based on projection from the day reached 100 cumulative cases to the day of RWD peak and RMSE for Scenario 5 was calculated based on projection from the day reaching 1600 cumulative cases to the day of RWD peak.

### Patient and public involvement

Patients or the public were not involved, and it was not appropriate or possible to involve patients or the public in the design, or conduct, or reporting, or dissemination plans of our research.

## Results

### Scenarios with different diagnosis rates

Supplementary Figs. 1 and 2 show our simulation of number of beds needed in the isolation ward and ICU from 15th December 2019 to 31st March 2020 in Wuhan. Heterogeneity existed between projection models based on parameter estimated from data collected during different stages of the epidemic. In Scenario 1, we estimated a daily new case rate of 0.0005%, daily removal rate of 6.3830% and ICU rate of 38% using data of the day reached 100 cumulative confirmed cases. Our model projected 1721 beds needed in isolation ward and ICU by 31st March 2020, assuming a 50% diagnosis rate. In Scenario 2, we used data collected from the period between 200 and 400 cumulative confirmed cases. The daily new case rate was estimated to be 0.0006%, which is close to that in Scenario 1; however, daily removal rate and ICU rate decreased to 1.9881% and 28%, respectively. Scenario 2 predicted a total of 4386 beds would be needed in isolation ward and ICU by the end of March in 2020. In general, as the epidemic developed, we observed an increasing daily new case rate, and decreasing daily removal rate and ICU rate. This change in rates contributed to the growth in the needs of bed in both isolation ward and ICU. However, such increasing trend reached a seemingly turning point between the periods of 800–1600 cumulative cases and 1600–3200 cumulative cases, which are reflected in Scenarios 5 and 6. Daily new case rate in Wuhan increased from 0.0019 to 0.0055%, and daily removal rate decreased from 1.7244 to 1.4368% in Scenario 5, followed by a drop from 0.0055 to 0.0037% in daily new case rate and increase from 1.4368 to 3.0341% in daily removal rate in Scenario 6. Also, the daily new case rate, the daily removal rate and the ICU rate of the additional scenario (parameters based on the day of 800–3200 cumulative confirmed cases) are 0.0046%, 1.3126 and 22% respectively.

### Scenarios with public health intervention and real-world data (RWD)

**Figure 2:**
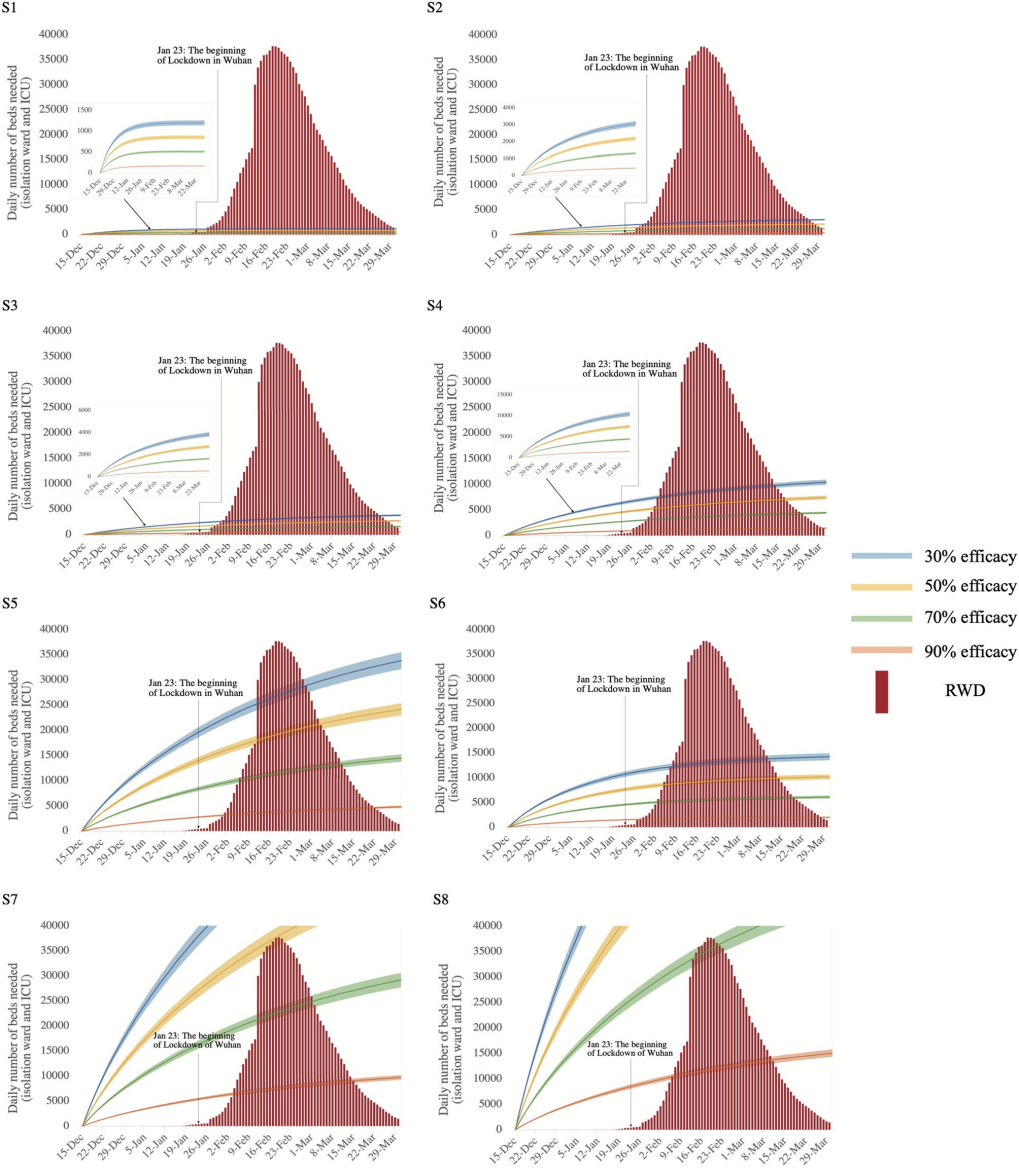
Comparison between early-stage SIR projection and real-world data (RWD) (assuming 50% diagnosis rate). *Notes* Scenario 1: parameter estimation based on data from the day when cumulative confirmed cases reached 100; Scenario 2: parameter estimation based on data between the day of 100 and the day of 200 cumulative confirmed cases; Scenario 3: parameter estimation based on data between the day of 200 and the day of 400 cumulative confirmed cases; Scenario 4: parameter estimation based on data between the day of 400 and the day of 800 cumulative confirmed cases; Scenario 5: parameter estimation based on data between the day of 800 and the day of 1600 cumulative confirmed cases; Scenario 6: parameter estimation based on data between the day of 1600 and the day of 3200 cumulative confirmed cases; Scenario 7: parameter estimation based on data between the day of 3200 and the day of 6400 cumulative confirmed cases; Scenario 8: parameter estimation based on data between the day of 6400 and the day of 12,800 cumulative confirmed cases.

NHC of People’s Republic of China began to release the epidemic data from 10th January 2020. RWD shows that the daily number of beds occupied in Wuhan’s hospitals due to COVID-19 increased from 10th January 2020, peaked on 18th February 2020 at 37,746, and dropped to 1456 by 30th March 2020 (Fig. 2). To compare our analysis to the RWD, we considered a 50% diagnosis rate and applied different public health intervention efficacies (30%, 50%, 70%, and 90%) to each scenario (Fig. 2). And the SIR projection based on data obtained during different stages of the epidemic (Starting from the day of which data were used for parameter estimation in each scenario to the day of real-world data (RWD) peak) has been shown in the Supplementary Table 2. In the scenarios where parameters were estimated based on data collected before 800 cumulative cases (i.e., Scenarios 1–4), the simulation largely underestimated the number of beds needed during the epidemic. However, Scenario 5 with 30% and 50% public health efficacy, Scenario 6 with 30% public health efficacy, Scenario 7 with 50% and 70% public health efficacy, and Scenario 8 with 70% public health efficacy returned a projection closer to the RWD peak. In general, RMSEs showed a decreasing trend as we used more updated data to estimate our model input (Table 2). In Scenarios 7 and 8, we also found a decreasing trend of RMSEs when public health efficacy increased from 30 to 70%.

**Table 2:** Number of beds needed as at the real-world data (RWD) peak and root mean square errors (RMSEs) of all scenarios.

Scenarios	Public health interventions							
	30%		50%		70%		90%	
	Beds needed at the RWD peak	RMSE^a^	Beds needed at the RWD peak	RMSE^a^	Beds needed at the RWD peak	RMSE^a^	Beds needed at the RWD peak	RMSE^a^
Scenario 1	1191	17,012	851	17,224	510	17,440	170	17,660
Scenario 2	2574	16,749	1839	17,192	1103	17,648	368	18,115
Scenario 3	3145	16,988	2247	17,541	1348	18,111	449	18,698
Scenario 4	8526	15,377	6090	16,799	3654	18,378	1218	20,075
Scenario 5	26,786	13,755	19,133	12,118	11,480	14,145	3827	18,679
Scenario 6	12,995	14,127	9282	16,216	5569	18,745	1856	21,561
Scenario 7	52,764	27,663	37,689	15,558	22,613	10,727	7538	19,603
Scenario 8	82,278	51,969	58,770	30,268	35,262	11,301	11,754	18,317

^a^RMSE for Scenario 1 was calculated based on projection from the day reached 100 cumulative cases to the day of RWD peak, for Scenario 2 was calculated based on projection from the day 200 cumulative cases to the day of RWD peak, for Scenario 3 was calculated based on projection from the day 400 cumulative cases to the day of RWD peak, for Scenario 4 was calculated based on projection from the day 800 cumulative cases to the day of RWD peak, for Scenario 5 was calculated based on projection from the day 1600 cumulative cases to the day of RWD peak, for Scenario 6 was calculated based on projection from the day 3200 cumulative cases to the day of RWD peak, for Scenario 7 was calculated based on projection from the day 6400 cumulative cases to the day of RWD peak, for Scenario 8 was calculated based on projection from the day 12,800 cumulative cases to the day of RWD peak.

## Discussion

In this study, we synthesized publicly available data from NHC of People’s Republic of China and Health Commission of Hubei Province during the early stage (mid-December 2019 to the end of March 2020) of COVID-19 epidemic in Wuhan. We constructed a modified SIR model based on parameters estimated using data at different stages of the epidemic, and projected daily number of beds needed. We acknowledge that a single model with time-varying parameters may be used to show the overall evolution of the outbreak; however, in this study we try to mimic the situation during a novel outbreak, when data and knowledge on the infection are limited. We design the scenarios based on the number of cumulative cases because this is what will be observed by the infectious disease control authority. In other words, this reflects the real situation during the early stage of the outbreak.

By comparing our model to RWD, we found that very early-stage projection (Scenarios 1–4) can be largely affected by the model input based on limited data. However, as the epidemic progressed and more data were available, SIR projection (Scenarios 5–8) showed better performance. The RMSEs showed that, in general, Scenario 1–4 have higher RMSEs than Scenario 5–8, except that, Scenarios 7 and 8 with 30% and 50% efficacy of public health intervention had RMSEs higher than the corresponding Scenarios 5 and 6. Additionally, RMSEs of Scenarios 7 and 8 increased as efficacy of public health intervention increased from 30 to 70%, which may suggest the governmental measures and public health intervention achieved a relatively high level in these time periods. Also, we found that estimation of 90% efficacy of public health intervention generally have high RMSEs, which may indicate that a 90% efficacy of public health intervention is too optimistic.

During the epidemic of a novel infectious disease, revision of diagnosis criteria, expanding of testing capacity, resource allocation, and newly implemented government measures and interventions, etc. can influenced data availability and updates, which affects analyses such as our SIR projection. The unexpected trend in Scenarios 5 and 6 using data from the period with 800–1600 and 1600–3200 cumulative cases respectively may be explained by the abovementioned factors. Specifically, on 27th Jan 2020, the cumulative number of cases jumped from 698 to 1590, we found it closely related to the new policies introduced, which directly resulted in that individuals with symptoms of COVID-19 getting tested timely. First, the government’s requirements of “early detection, early isolation, early reporting, and early treatment” was further implemented in Wuhan from 18th January 2020^28^. Second, more resources had been allocated to assist the screening of fever patients, 24-h consultations at fever clinics, and accelerated testing, etc. Third, direct reporting by medical institutions to NHC also improved the efficiency of data sharing and information updates. Following this sharp increase in the number of cases, daily removal rate in Scenario 6 almost doubled comparing to Scenario 5 (Supplementary Table 1), this may be due to the nation-wide effort in sending medical equipment and personnel from all around China to Wuhan, most of whom arrived between 28th January 2020 and 29th January 2020^29^. However, daily removal rate dropped immediately in Scenarios 7, implying a higher uncertainty in the projection of Scenario 6. Taken together, mathematical modelling such as SIR model could be an effective and useful tool to provide guidance about the infectious disease containing measures on the early stage of the epidemic. Furthermore, our study shows that despite its simplicity, the SIR model still has good accuracy and can predict general epidemic trends. This also implies that in the early stage of the spread of new infectious diseases in the future, the use of this simple SIR model will be convenient and effective in predicting the possibility and severity of disease outbreaks, and timely provide key information for the public health system. Nevertheless, model inputs should be frequently revisited considering the fluctuation of early-stage data and the impacts of policy-related factors should be reviewed cautiously.

Our analysis highlights the importance of achieving a timely and effective public health intervention during the course of epidemic. In Wuhan, various non-pharmaceutical interventions (NPIs) to significantly reduce the transmission of SARS-CoV-2 and human-to-human contacts were implemented after the outbreak, including the lockdown in Wuhan on 23rd January 2020, mandatory face masks wearing, social distancing policies^19^, etc. At the same time, Chinese government and Wuhan government also made some other considerable efforts to relief the demand for hospital beds in isolation ward and ICU, including the construction of the first Wuhan field hospital^30^ (Wuhan Huoshenshan Hospital) and open on 3rd February 2020, and later the second Wuhan field hospital^31^ (Wuhan Leishenshan Hospital) open on 8th February 2020. With the strong and effective intervention, Wuhan achieved its success in containing the pandemic, the hospital beds occupied declined evidently, and the daily new case declined to 0 on 18th March 2020. Additionally, our study highlighted and gave an important public health message that, the latent hazards of waiting for a complicated model (e.g., extra deaths) and delay-decision may be far greater than the benefits at the very-early stage of the outbreaks (i.e., January–March of 2021), although complicated models may provide more insights and better estimation and simulation. Therefore, the present study highlighted that early-stage simple model has the potential to show the scale of the outbreak and urge the governments to take immediate action. Only with the initial public health measures (including active data collection), can more complicated models be constructed. In addition, early action may save numerous lives by providing a sufficient number of hospital beds, which is another focus of our work. We acknowledge that evidence-based policymaking is necessary; however, we should not expect policymaking to be based on perfect or near-perfect modelling. Therefore, early-stage simple model helps policymakers to avoid issue of delay-decision at the very-early stage of the outbreak and take the initiative to hinder extra deaths using very-early-stage data. While these messages have not been discussed in the previous studies, and thus indicate the implacable of this study.

Our study also highlighted that the effective method to control the infectious diseases outbreaks at the very-early stage cannot solely rely on the prediction of the trend of the outbreaks, while policymakers and medical system managers should also actively collaborate with medical agencies to effectively allocate a sufficient number of hospital beds to ensure timely treatment for patients. While it is also important to refer to the latest data in a timely manner. Public health policies require the awareness of planning remedial measures and resources in advance and setting up departments and mechanisms incorporation such as crisis management.

Some limitations exist in this study. First, several assumptions were made to construct an SIR model. Populations are divided into three compartments and the transmission process of the disease may not reflect the real situation and thus may be oversimplified to some extent. For example, latency period^32^ to the infectious transition between susceptible status and infectious status is not considered in the model in view of the model simplicity. Also, the constant total population and several rates between different compartments were assumed, such as constant contact rate and constant fraction rate for infectious compartment individuals to remove. However, with limited data and information at the early stage of an epidemic, overcomplicated model may neither serve well. Second, the SIR model assumes a closed and homogeneous mixing population^32^, which means that the probability of people’s contacting is equal, which is unrealistic. Third, the SIR model simulation largely relies on the availability and update from the government webpage such as NHC and CDC, especially the RWD. Fourth, knowledge about COVID-19 may be very limited in the early stage, and the change of case definition^33^ exists throughout the epidemic, and potentially resulted in the prediction bias, therefore, it is essential incorporating data from various sources, including clinical and epidemiological data, to improve the accuracy of the model. Last but not least, the applicability of the SIR model is typically limited at the very-early stage of the outbreak, since it is very natural and reasonable to reconstruct more accurate dynamics (if we assume most of the infected individuals were confirmed or assumed the constant under-ascertainment rate over time), uncertainty during the early stage of the disease outbreak may introduce bias into the infectious disease model, which may thereby lead to less effective public health control measures, while we still can convert this limitation to an opportunity to reduce the extra deaths and avoid delay-decision issue, using very-early-stage epidemiological and clinical data. Since, for example, in January to March of 2021 in Wuhan China, high case-fatality rate (CFR) of wild-type SARS-CoV-2 is relatively higher, and there were very restricted information on wild-type SARS-CoV-2; even in January of 2021, the transmission mode and incubation rate of the virus also remains unknown. Thus, complicated models for dynamic prediction may not show a good performance due to limited parameters feeding into the model. In contrast, a simple model is very convenient and has a relatively low requirement for the number of parameters.

## Conclusion

Our study indicates that at the very-early stage of the infectious disease, the SIR model performs relatively soundly and accurately, so policymakers can avoid delay-decision, and many countries’ public health systems can avoid breakdown. However, due to the nature of the SIR model, when we know more about the diseases and have more epidemiological data, a more complicated model may typically provide a better estimation and prediction of the epidemic.

## Supplementary Information

Supplementary Information.

## Data Availability

All data relevant to this study are included in the article and supplemental information. Some data were acquired from the National Health Commission of the People's Republic of China, from the website of: http://www.nhc.gov.cn/xcs/yqtb/list_gzbd.shtml.
