# Supplementary material for: A real-world data validation of the value of early-stage SIR modelling to public health
Source: Sci Rep. 2023 Jun 6;13:9164. doi: 10.1038/s41598-023-36386-9 (PMC10242229; doi:10.1038/s41598-023-36386-9)
Supplement: Supplementary file 1 — Supplementary Information. [file 41598_2023_36386_MOESM1_ESM.pdf]

Supplemental material

Supplementary Table 1. Settings of time intervals and model inputs of all scenarios

Scenarios	Time intervals		Model inputs							
	Starts	Ends	Number of daily new confirmed cases (Wuhan)	Number of daily existing confirmed cases (Wuhan)	Number of daily existing confirmed cases (Hubei)	Number of daily existing ICU (Hubei)	Number of Daily removal (Hubei)	DailyNewCase.Rate (Based on Wuhan)	DailyRemoval.Rate (Based on Hubei)	ICU.Rate (Based on Hubei)
Scenario 1	The day with 100 cumulative cases	The day with 100 cumulative cases	59	94	94	36	6	0.0005%	6.3830%	38%
Scenario 2	The day with 100 cumulative cases	The day with 200 cumulative cases	65	164	168	48	3	0.0006%	1.9881%	28%
Scenario 3	The day with 200 cumulative cases	The day with 400 cumulative cases	76	312	325	82	6	0.0007%	1.7418%	25%
Scenario 4	The day with 400 cumulative cases	The day with 800 cumulative cases	205	651	1063	248	18	0.0019%	1.7244%	23%
Scenario 5	The day with 800 cumulative cases	The day with 1600 cumulative cases	604	1590	2958	794	43	0.0055%	1.4437%	27%
Scenario 6	The day with 1600 cumulative cases	The day with 3200 cumulative cases	406	2254	4977	1069	151	0.0037%	3.0341%	21%
Scenario 7	The day with 3200 cumulative cases	The day with 6400 cumulative cases	1142	4923	10845	1844	136	0.0104%	1.2522%	17%
Scenario 8	The day with 6400 cumulative cases	The day with 12800 cumulative	1805	9960	19495	3700	251	0.0164%	1.2875%	19%

Supplementary Table 2. SIR projection based on data obtained during different stages of the epidemic (starting from the day of which data were used for parameter estimation in each scenario to the day of real-world data (RWD) peak)

Date	RWD	Scenarios							
		Scenario 1 (50% Dx.Rate)				Scenario 2 (50% Dx.Rate)			
		30%	50%	70%	90%	30%	50%	70%	90%
**18-Jan**	94	1088	777	466	155				
**19-Jan**	170	1095	782	469	156				
**20-Jan**	228	1103	788	473	158	1883	1345	807	269
**21-Jan**	327	1109	792	475	158	1914	1367	820	273
**22-Jan**	381	1115	797	478	159	1945	1389	834	278
**23-Jan**	441	1121	801	481	160	1975	1411	846	282
**24-Jan**	502	1127	805	483	161	2004	1432	859	286
**25-Jan**	533	1132	808	485	162	2033	1452	871	290
**26-Jan**	593	1137	812	487	162	2061	1472	883	294
**27-Jan**	1458	1141	815	489	163	2089	1492	895	298
**28-Jan**	1721	1145	818	491	164	2116	1512	907	302
**29-Jan**	2045	1149	821	493	164	2143	1531	918	306
**30-Jan**	2372	1153	823	494	165	2169	1549	929	310
**31-Jan**	2879	1156	826	496	165	2194	1567	940	313
**1-Feb**	3709	1160	828	497	166	2219	1585	951	317
**2-Feb**	4648	1163	830	498	166	2244	1603	962	321
**3-Feb**	5763	1165	832	499	166	2268	1620	972	324
**4-Feb**	7616	1168	834	501	167	2291	1636	982	327
**5-Feb**	9267	1170	836	502	167	2314	1653	992	331
**6-Feb**	10601	1173	838	503	168	2337	1669	1001	334
**7-Feb**	12355	1175	839	503	168	2359	1685	1011	337
**8-Feb**	13492	1177	841	504	168	2380	1700	1020	340
**9-Feb**	15173	1179	842	505	168	2401	1715	1029	343
**10-Feb**	16496	1180	843	506	169	2422	1730	1038	346
**11-Feb**	17356	1182	844	507	169	2443	1745	1047	349
**12-Feb**	30038	1184	845	507	169	2463	1759	1055	352
**13-Feb**	33490	1185	846	508	169	2482	1773	1064	355
**14-Feb**	34820	1186	847	508	169	2501	1787	1072	357
**15-Feb**	35953	1188	848	509	170	2520	1800	1080	360
**16-Feb**	36111	1189	849	510	170	2538	1813	1088	363
**17-Feb**	36878	1190	850	510	170	2557	1826	1096	365
**18-Feb**	37746	1191	851	510	170	2574	1839	1103	368

(To be continued)

Date	RWD								
		Scenario 3 (50% Dx.Rate)				Scenario 4 (50% Dx.Rate)			
		30%	50%	70%	90%	30%	50%	70%	90%
**18-Jan**	94								
**19-Jan**	170								
**20-Jan**	228								
**21-Jan**	327								
**22-Jan**	381	2325	1661	997	332				
**23-Jan**	441	2363	1688	1013	338				
**24-Jan**	502	2401	1715	1029	343				
**25-Jan**	533	2437	1741	1045	348				
**26-Jan**	593	2473	1767	1060	353				
**27-Jan**	1458	2509	1792	1075	358	6793	4852	2911	970
**28-Jan**	1721	2543	1817	1090	363	6888	4920	2952	984
**29-Jan**	2045	2577	1841	1105	368	6980	4986	2991	997
**30-Jan**	2372	2611	1865	1119	373	7071	5051	3030	1010
**31-Jan**	2879	2644	1888	1133	378	7160	5115	3069	1023
**1-Feb**	3709	2676	1911	1147	382	7248	5177	3106	1035
**2-Feb**	4648	2708	1934	1160	387	7334	5239	3143	1048
**3-Feb**	5763	2739	1956	1174	391	7419	5299	3180	1060
**4-Feb**	7616	2769	1978	1187	396	7502	5359	3215	1072
**5-Feb**	9267	2799	2000	1200	400	7584	5417	3250	1083
**6-Feb**	10601	2829	2021	1212	404	7664	5475	3285	1095
**7-Feb**	12355	2858	2041	1225	408	7743	5531	3319	1106
**8-Feb**	13492	2886	2062	1237	412	7821	5586	3352	1117
**9-Feb**	15173	2914	2082	1249	416	7897	5641	3384	1128
**10-Feb**	16496	2942	2101	1261	420	7972	5694	3417	1139
**11-Feb**	17356	2969	2121	1272	424	8045	5747	3448	1149
**12-Feb**	30038	2995	2140	1284	428	8118	5798	3479	1160
**13-Feb**	33490	3022	2158	1295	432	8189	5849	3509	1170
**14-Feb**	34820	3047	2177	1306	435	8258	5899	3539	1180
**15-Feb**	35953	3072	2195	1317	439	8327	5948	3569	1190
**16-Feb**	36111	3097	2212	1327	442	8394	5996	3598	1199
**17-Feb**	36878	3121	2230	1338	446	8461	6043	3626	1209
**18-Feb**	37746	3145	2247	1348	449	8526	6090	3654	1218

(To be continued)

Date	RWD	Scenario 5 (50% Dx.Rate)				Scenario 6 (50% Dx.Rate)			
		30%	50%	70%	90%	30%	50%	70%	90%
**18-Jan**	94								
**19-Jan**	170								
**20-Jan**	228								
**21-Jan**	327								
**22-Jan**	381								
**23-Jan**	441								
**24-Jan**	502								
**25-Jan**	533								
**26-Jan**	593								
**27-Jan**	1458								
**28-Jan**	1721	21214	15153	9092	3031				
**29-Jan**	2045	21521	15372	9223	3074				
**30-Jan**	2372	21823	15588	9353	3118				
**31-Jan**	2879	22120	15800	9480	3160	11652	8323	4994	1665
**1-Feb**	3709	22414	16010	9606	3202	11749	8392	5035	1678
**2-Feb**	4648	22702	16216	9730	3243	11842	8458	5075	1692
**3-Feb**	5763	22987	16419	9852	3284	11932	8523	5114	1705
**4-Feb**	7616	23267	16620	9972	3324	12020	8585	5151	1717
**5-Feb**	9267	23544	16817	10090	3363	12104	8646	5188	1729
**6-Feb**	10601	23816	17011	10207	3402	12187	8705	5223	1741
**7-Feb**	12355	24084	17203	10322	3441	12266	8762	5257	1752
**8-Feb**	13492	24348	17392	10435	3478	12343	8817	5290	1763
**9-Feb**	15173	24609	17578	10547	3516	12418	8870	5322	1774
**10-Feb**	16496	24865	17761	10656	3552	12491	8922	5353	1784
**11-Feb**	17356	25118	17941	10765	3588	12561	8972	5383	1794
**12-Feb**	30038	25367	18119	10871	3624	12629	9021	5412	1804
**13-Feb**	33490	25612	18294	10977	3659	12695	9068	5441	1814
**14-Feb**	34820	25854	18467	11080	3693	12759	9113	5468	1823
**15-Feb**	35953	26092	18637	11182	3727	12820	9157	5494	1831
**16-Feb**	36111	26327	18805	11283	3761	12880	9200	5520	1840
**17-Feb**	36878	26558	18970	11382	3794	12939	9242	5545	1848
**18-Feb**	37746	26786	19133	11480	3827	12995	9282	5569	1856

(To be continued)

Date	RWD	Scenario 7 (50% Dx.Rate)				Scenario 8 (50% Dx.Rate)			
		30%	50%	70%	90%	30%	50%	70%	90%
**18-Jan**	94								
**19-Jan**	170								
**20-Jan**	228								
**21-Jan**	327								
**22-Jan**	381								
**23-Jan**	441								
**24-Jan**	502								
**25-Jan**	533								
**26-Jan**	593								
**27-Jan**	1458								
**28-Jan**	1721								
**29-Jan**	2045								
**30-Jan**	2372								
**31-Jan**	2879								
**1-Feb**	3709								
**2-Feb**	4648								
**3-Feb**	5763								
**4-Feb**	7616	45448	32463	19478	6493				
**5-Feb**	9267	46017	32869	19721	6574				
**6-Feb**	10601	46578	33270	19962	6654				
**7-Feb**	12355	47131	33665	20199	6733	73661	52615	31569	10523
**8-Feb**	13492	47678	34055	20433	6811	74499	53214	31928	10643
**9-Feb**	15173	48217	34441	20664	6888	75326	53804	32283	10761
**10-Feb**	16496	48749	34821	20892	6964	76142	54387	32632	10877
**11-Feb**	17356	49274	35196	21118	7039	76946	54962	32977	10992
**12-Feb**	30038	49793	35566	21340	7113	77740	55528	33317	11106
**13-Feb**	33490	50304	35932	21559	7186	78522	56087	33652	11217
**14-Feb**	34820	50809	36292	21775	7258	79294	56639	33983	11328
**15-Feb**	35953	51308	36648	21989	7330	80056	57183	34310	11437
**16-Feb**	36111	51800	37000	22200	7400	80807	57719	34631	11544
**17-Feb**	36878	52285	37347	22408	7469	81547	58248	34949	11650
**18-Feb**	37746	52764	37689	22613	7538	82278	58770	35262	11754

30%, 50%, 70%, 90% represent 30%, 50%, 70%, 90% efficacy of public health intervention

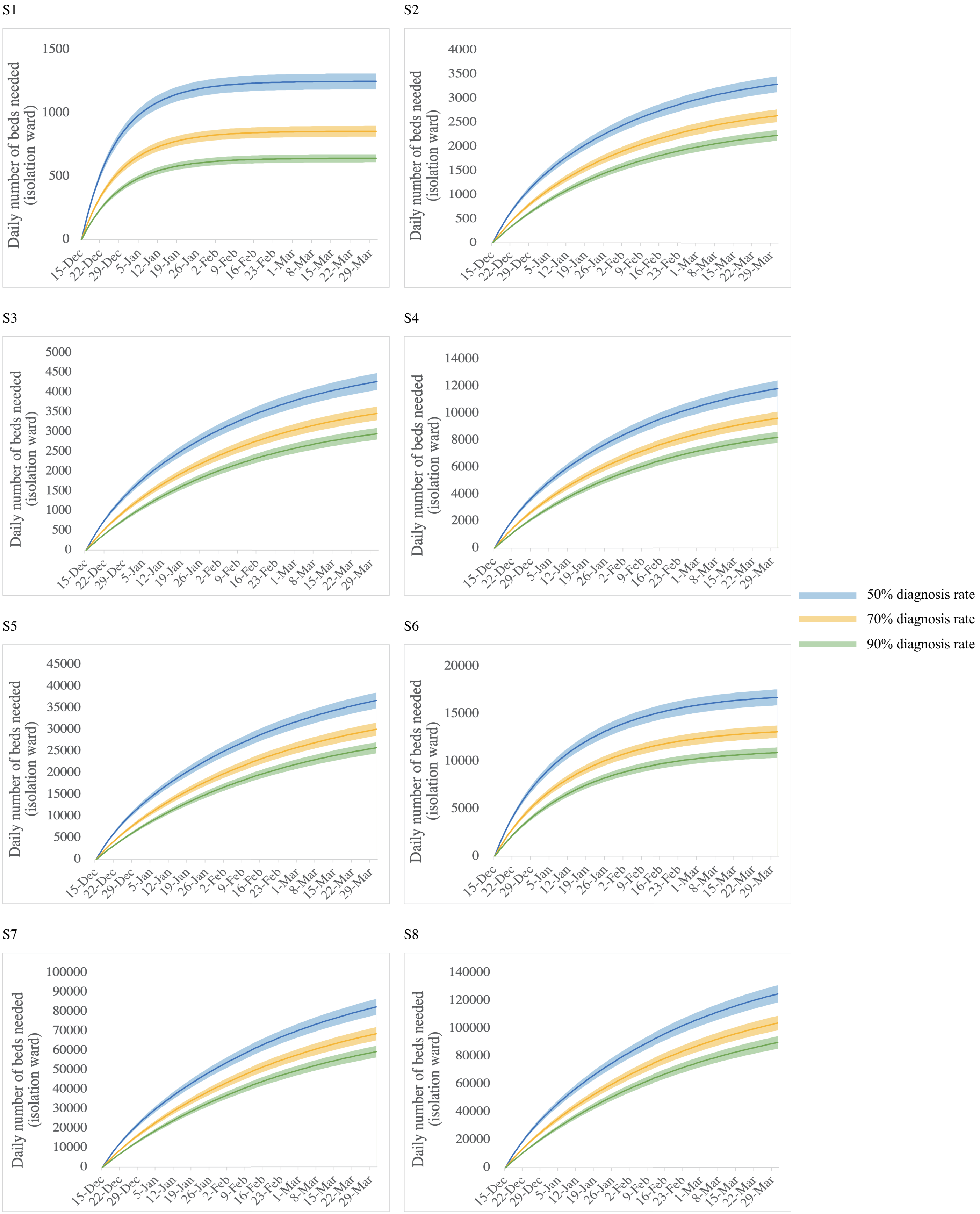

Supplementary Figure 1. **Simulated daily number of beds needed in isolation ward in Wuhan with different diagnosis rate.** (The blue line represents 50% diagnosis rate, the yellow line represents 70% diagnosis rate and the green line represents 90% diagnosis rate. Scenario 1: parameter estimation based on data from the day when cumulative confirmed cases reached 100; Scenario 2: parameter estimation based on data between the day of 100 and the day of 200 cumulative confirmed cases; Scenario 3: parameter estimation based on data between the day of 200 and the day of 400 cumulative confirmed cases; Scenario 4: parameter estimation based on data between the day of 400 and the day of 800 cumulative confirmed cases; Scenario 5: parameter estimation based on data between the day of 800 and the day of 1600 cumulative confirmed cases; Scenario 6: parameter estimation based on data between the day of 1600 and the day of 3200 cumulative confirmed cases; Scenario 7: parameter estimation based on data between the day of 3200 and the day of 6400 cumulative confirmed cases; Scenario 8: parameter estimation based on data between the day of 6400 and the day of 12800 cumulative confirmed cases.)

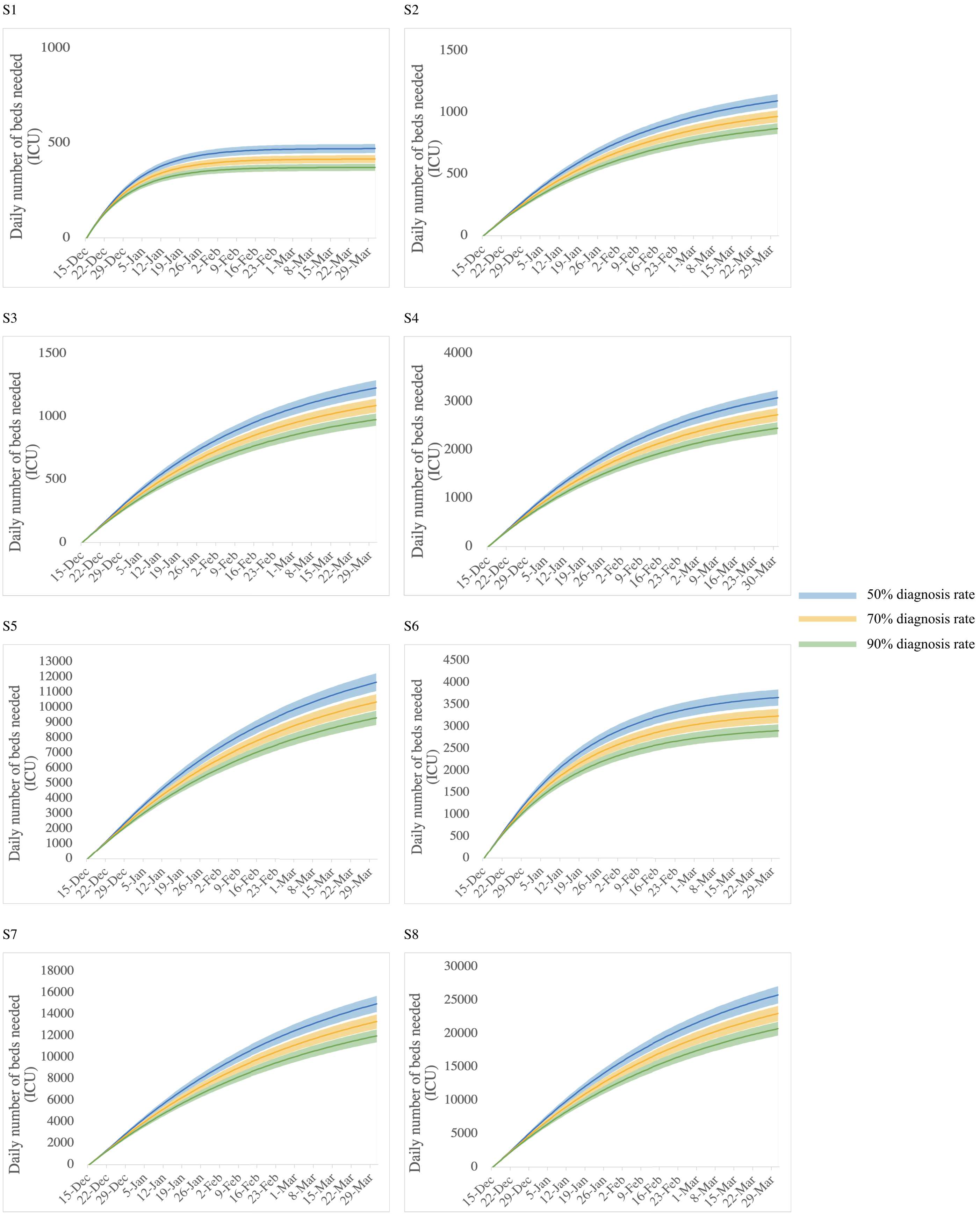

Supplementary Figure 2. **Simulated daily number of beds needed in intensive care unit (ICU) in Wuhan with different diagnosis rate.** (The blue line represents 50% diagnosis rate, the yellow line represents 70% diagnosis rate and the green line represents 90% diagnosis rate. Scenario 1: parameter estimation based on data from the day when cumulative confirmed cases reached 100; Scenario 2: parameter estimation based on data between the day of 100 and the day of 200 cumulative confirmed cases; Scenario 3: parameter estimation based on data between the day of 200 and the day of 400 cumulative confirmed cases; Scenario 4: parameter estimation based on data between the day of 400 and the day of 800 cumulative confirmed cases; Scenario 5: parameter estimation based on data between the day of 800 and the day of 1600 cumulative confirmed cases; Scenario 6: parameter estimation based on data between the day of 1600 and the day of 3200 cumulative confirmed cases; Scenario 7: parameter estimation based on data between the day of 3200 and the day of 6400 cumulative confirmed cases; Scenario 8: parameter estimation based on data between the day of 6400 and the day of 12800 cumulative confirmed cases.)

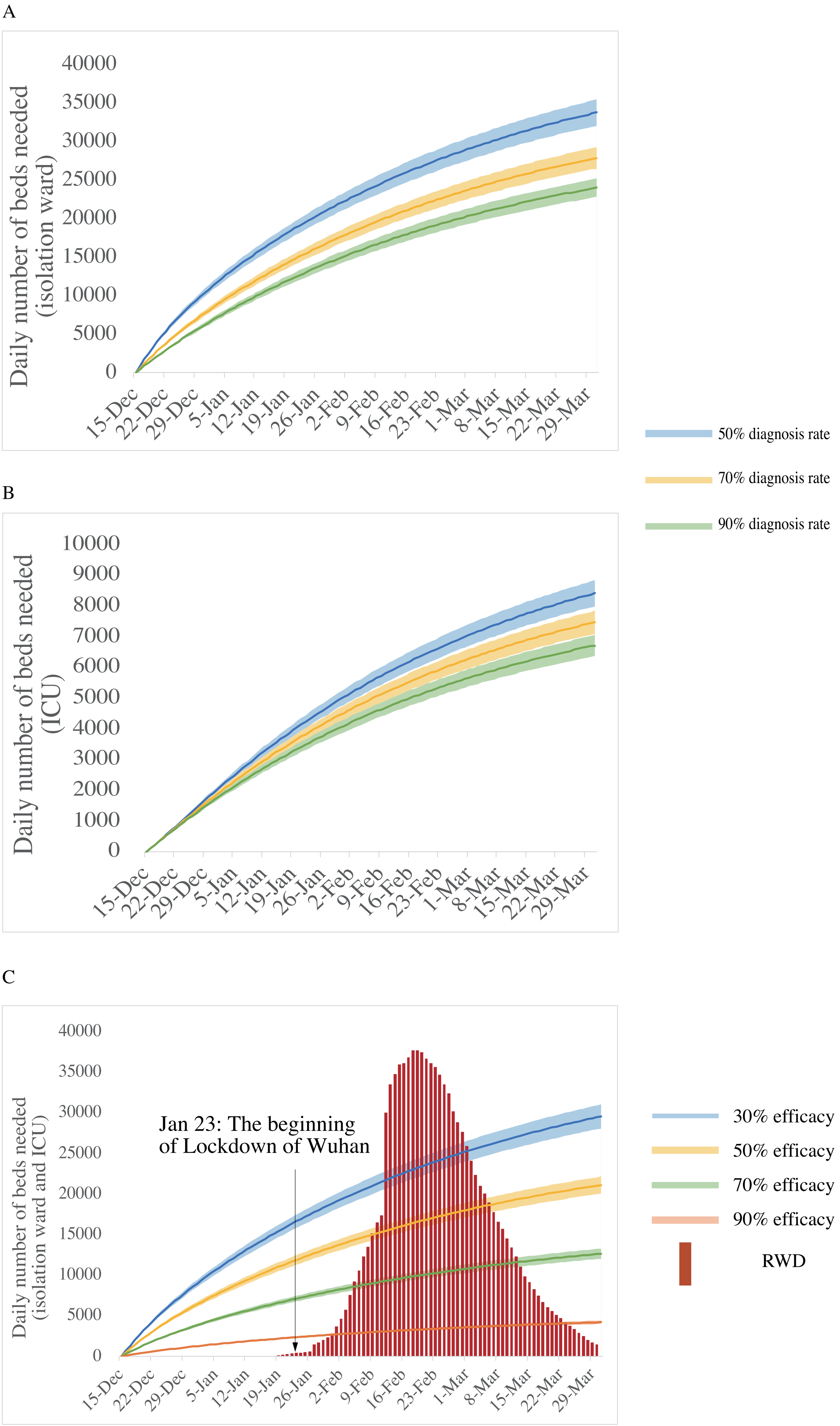

Supplementary Figure 3. Scenario based on parameter estimated using data from the day reaching 800 cumulative cases to the day reaching 3200 cumulative cases.

(A. Beds needed in isolation ward with 50%, 70% and 90% diagnosis rates and no public health intervention. B. Beds needed in ICU with 50%, 70% and 90% diagnosis rates and no public health intervention. C. Beds needed with 30%, 50%, 70% and 90% public health intervention assuming 50% diagnosis rate, compared with RWD).
